# Modification of the RUSLE slope length and steepness factor (LS-factor) based on rainfall experiments at steep alpine grasslands

**DOI:** 10.1016/j.mex.2019.01.004

**Published:** 2019-01-26

**Authors:** Simon Schmidt, Simon Tresch, Katrin Meusburger

**Affiliations:** a Environmental Geosciences, University of Basel, Bernoullistrasse 30, CH-4056 Basel, Switzerland; b Department of Soil Sciences, Research Institute of Organic Agriculture (FiBL), Ackerstrasse 113, CH-5070 Frick, Switzerland; c Functional Ecology Laboratory, Institute of Biology, University of Neuchâtel, Rue Emile-Argand 11, CH- 2000 Neuchâtel, Switzerland; d Biodiversity and Conservation Biology, Swiss Federal Institute for Forest, Snow and Landscape Research (WSL), Zürcherstrasse 111, CH-8903 Birmensdorf, Switzerland; e Forest Soils and Biogeochemistry, Swiss Federal Institute for Forest, Snow and Landscape Research (WSL), Zürcherstrasse 111, CH-8903 Birmensdorf, Switzerland

**Keywords:** L_alpine_, S_alpine_, LS_alpine_, Revised Universal Soil Loss Equation, Erosion modeling, Switzerland, Terrain features, Maximal, Flow length

## Abstract

The slope length and slope steepness factor (LS-factor) is one of five factors of the Universal Soil Loss Equation (USLE) and its revised version (RUSLE) describing the influence of topography on soil erosion risk. The LS-factor was originally developed for slopes less than 50% inclination and has not been tested for steeper slopes. To overcome this limitation, we adapted both factors slope length L and slope steepness S for conditions experimentally observed at Swiss alpine grasslands. For the new L-factor (L_alpine_), a maximal flow path threshold, corresponding to 100 m, was implemented to take into account short runoff flow paths and rapid infiltration that has been observed in our experiments. For the S-factor, a fitted quadratic polynomial function (S_alpine_) has been established, compiling the most extensive empirical studies. As a model evaluation, uncertainty intervals are presented for this modified S-factor. We observed that uncertainty increases with slope gradient. In summary, the proposed modification of the LS-factor to alpine environments enables an improved prediction of soil erosion risk including steep slopes.

• Empirical experiments (rainfall simulation, sediment measurements) were conducted on Swiss alpine grasslands to assess the maximal flow length and slope steepness factor (S-factor).
• Flow accumulation is limited to a maximal flow threshold (100 m) at which overland runoff is realistic in alpine grassland.
• Slope steepness factor is modified by a fitted S-factor equation from existing empirical S-factor functions.

## Graphical abstract

**Figure:**
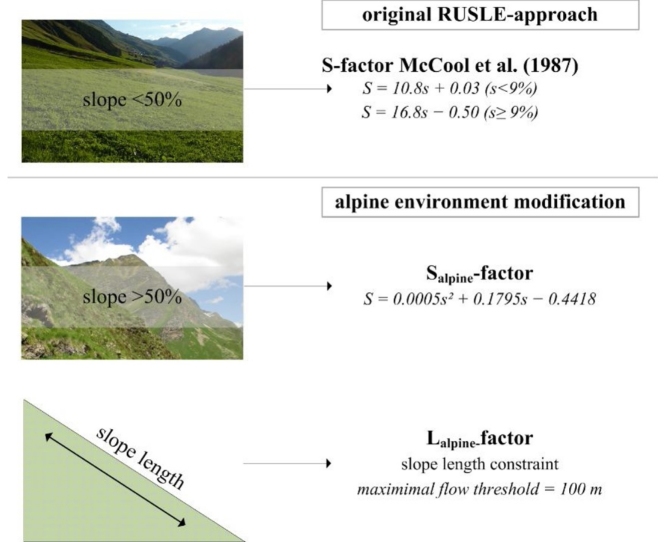

**Specifications Table**

**Table:** 

Subject area	*Environmental Science*
More specific subject area	*Soil erosion modeling*
Method name	- *L_alpine_* - *S_alpine_* - *LS_alpine_*
Name and reference of original method	USLE LS-factor: Wischmeier, W.H., & Smith, D.D. (1978). Predicting rainfall erosion losses. Washington.
	S-factor: McCool, D.K., Brown, L.C., Foster, G.R., Mutchler, C.K., & Meyer, L.D. (1987). Revised Slope Steepness Factor for the Universal Soil Loss Equation. Transactions of the ASAE, 30, 1387–1396. https://doi.org/doi:10.13031/2013.30576.
	S-factor: Smith, D.D., & Whitt, D. (1948). Estimating soil losses from field areas. Agricultural Engineering, 29, 394–396.
Resource availability	- *SAGA GIS* (http://www.saga-gis.org; [31]) - *RSAGA* (https://cran.r-project.org/web/packages/RSAGA/index.html; [30])

## Method details

### Existing approaches for S- and L-factor parametrization

The LS-factor is a product of the slope length (L-) and the slope steepness (S-factor). The most widely used slope length factor represents the ratio of observed soil loss related to the soil loss of a standardized plot (22.13 m). Originally Wischmeier and Smith [1] defined the L-factor as Eq. (1):

(1) $$L={\left(\frac{\lambda }{22.13}\right)}^{m}$$

where λ represents the length of the slope in meters and m the different slope steepness. Later, Eq. (2) was adapted for the RUSLE-approach to better describe soil loss with increasing slope steepness. Desmet and Govers [2] transformed the original L-factor (Eq. (1)) into a GIS-approach (Eq. (2)) considering the flow accumulation and adding a ratio of rill to interrill erosion (Eq. (3)):

(2) $$L_{i,j}=\frac{{\left(A_{i,j-in\;}+D^{2}\right)}^{m+1}-A_{i,j-in}^{m+1}}{D^{m+2}\;*\;X_{i,j\;}^{m}*\;22.13^{m}}$$

where A_i_,j-_in_ is the flow accumulation in m² at the inlet of a grid cell (i,j). D is the grid cell size in m and $X_{i,j}$ equals to $\text{sin}a_{i,j}+\text{cos}a_{i,j}$ where a_i,j_ is the aspect of the grid cell (i,j). The coefficient m (Eq. (3)) represents the ratio of rill and interrill erosion and is calculated by the β-value (Eq. (4)):

(3) $$m=\frac{\beta }{\beta +1}$$

with a range between 0 (ratio of rill to interrill erosion close to 0) and 1.

(4) $$\beta =\frac{\frac{\text{sin}\theta }{0.0896}}{\left[0.56+3*{\left(\text{sin}\theta \right)}^{0.8}\right]}$$

where θ is the slope angle in degrees.

For the S-factor, most often the empiric function proposed by McCool et al. [3] is used to determine the slope steepness factor in the Revised Universal Soil Loss Equation (RUSLE). McCool et al. [3] differentiate the relation between soil loss and slope steepness in radians (s) with two functions. One for slopes with an inclination less than 9% and the other greater or equal 9%. The functions are as follows:

(5) S=10.8s+0.03   for  slope steepness  in  percent<9%

(6) S=16.8s−0.50  for slope steepness  in percent≥9%

The S-factor after McCool et al. [3] is particular recommended for areas with low summer rainfall amounts [4]. Many other empirical S-factors were developed since the 1940s (Table 1) but all S-factors have in common that empirical evidence and thus validity is limited to slope gradients less than 50%.

**Table 1:** Review of selected S-factors (S).

Source	function	Description
Zingg [5]	$S={\left(\frac{s}{9}\right)}^{1.4}$	s = slope steepness in percent
Musgrave [6]	$S={\left(\frac{s}{9}\right)}^{1.35}$	s = slope steepness in percent
Smith and Whitt [7]	$S=0.025+0.052s^{\frac{4}{3}}$	s = slope steepness in percent
Smith [8]	$S=0.00650s^{2}+0.0453s+0.065$	s = slope steepness in percent
Smith [8]	$S=0.044+0.10s-0.00073s^{2}$	s = slope steepness in percent
Wischmeier and Smith [1]	$S=65.4\text{sin}\theta^{2}+4.56\text{sin}\theta +0.0654$	θ = slope steepness in radians
McCool et al. [9]	$S={\left(\frac{\text{sin}\theta }{0.00896}\right)}^{0.6}$	θ = slope steepness in radians
Foster [10]	$S=3{\left(\text{sin}\theta \right)}^{0.8}+0.56$	θ = slope steepness in radians
McCool et al. [3]	S=16.8sinθ−0.5	θ = slope steepness in radians
McCool et al. [3]	S=10.8sinθ+0.03	θ = slope steepness in radians
Nearing [11]	$S=\;-1.5+\;\frac{17}{1+\;e^{2.3-6.1\text{sin}\theta }}$	θ = slope steepness in radians
Liu et al. [12]	S=21.91sinθ−0.96	θ = slope steepness in radians
S_alpine_ present study	$S=0.0005s^{2}+0.1795s-0.4418$	s = slope steepness in percent

### Proposed adaption of the L-factor

Often, GIS modeled potential flow path length on slopes, expressed as flow accumulation in a GIS-environment, is driven by gravity and theoretically unlimited [13]. In particular cases, these potential flow path lengths can reach many kilometers and enormous runoff volumes. The flow accumulation can be constrained by streets or houses as ending points of the potential flow paths as discussed by Winchell et al. [14].

**Fig. 1:**
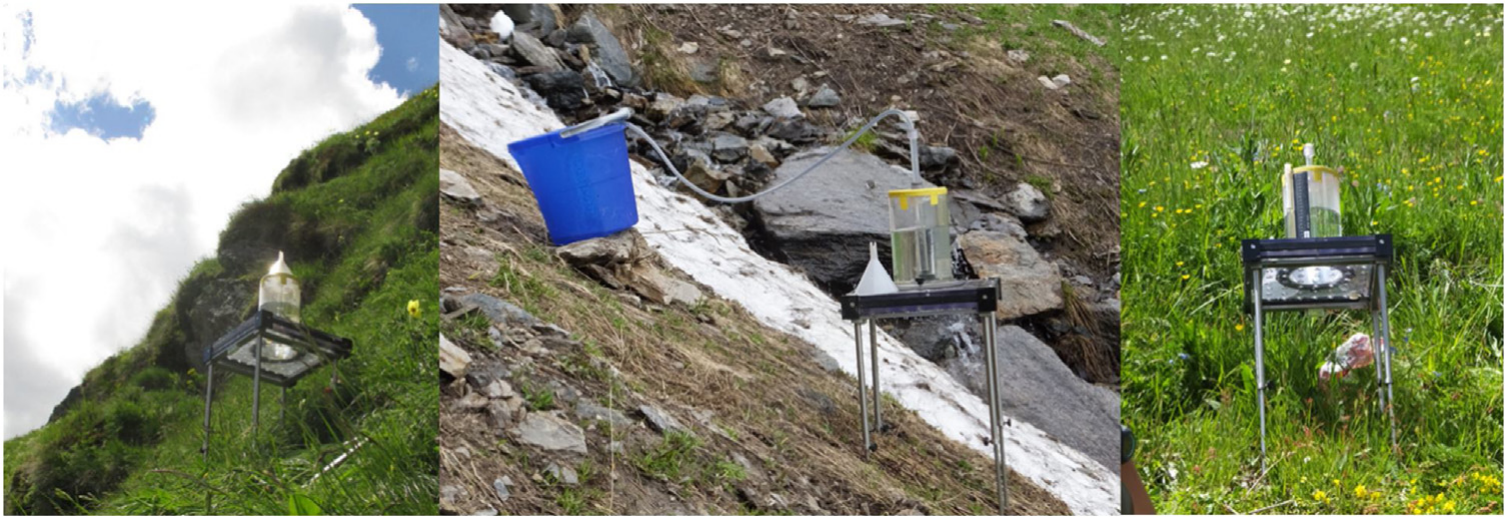
Different set ups and preconditions of the rainfall simulation experiment on steep slopes in Val Piora, Ticino, Switzerland.

In 2016, we conducted 19 different rainfall simulation experiments on south facing slopes in an alpine environment (Val Piora, Switzerland) with different conditions regarding soil moisture (dry, moist), steepness (36°–82°), and vegetation (low, medium, full vegetation cover) to observe the flow path lengths. The rainfall simulations were realized with an Eijkelkamp mini rainfall simulator (type M1.09.06.E, Eijkelkamp, NL; Fig. 1) for erosion tests with a rainfall intensity of 640 mm/h and an energy of 4 J mm^−1^ m^−2^. This rainfall energy is comparable with the average rainfall energy of Val Piora (station Piotta; 5.6 J mm^−1^ m^−2^; [15]). Regardless of the conditions, our observations revealed short surface flow path lengths at the scale of meters with a rapid infiltration into shallow alpine soils (see Appendix A. supplementary material). Our measurements and observations show, that potential flow paths without considering infiltration is not realistic for alpine environments and thus, requesting a maximal flow threshold for the estimation of the slope length factor L. McCool et al. [16] and Winchell et al. [14] limited the slope length to a maximal threshold of 333 m (1000 feet) as longer slope length appear only occasionally. According to McCool et al. [16], the usual threshold in many cases is 121 m (400 feet). As a compromise of their suggestion and our observed short flow path lengths in the Swiss Alps, we decided to limit the maximal flow length to 100 m.

The threshold is implemented as a condition either directly in SAGA GIS or in RSAGA after creating the flow accumulation grid:

(7) $$A_{alpine\;i,j-in\;}=ifelse\left(A_{i,j-in\;}>thresh,\;thresh,\;A_{i,j-in\;}\right)$$

where A_alpine i_,j-_in_ is the constraint flow accumulation in m² at the inlet of a grid cell (i,j) considering a threshold value *thresh*. That constraint flow accumulation value is inserted into the L-factor equation for the alpine environment (Eq. (8)):

(8) $$L_{alpine\;i,j}=\frac{{\left(A_{alpine\;i,j-in\;}+D^{2}\right)}^{m+1}-A_{alpine\;i,j-in}^{m+1}}{D^{m+2}\;\text{*}\;X_{i,j\;}^{m}\text{*}\;22.13^{m}}$$

Likewise to Eq. (2), D is the grid cell size in m and $X_{i,j}$ equals to $\text{sin}a_{i,j}+\text{cos}a_{i,j}$ where a_i,j_ is the aspect of the grid cell (i,j). The coefficient m is the ratio of rill (β-value) to interrill erosion according to the above mentioned Eqs. (3) and (4).

**Fig. 2:**
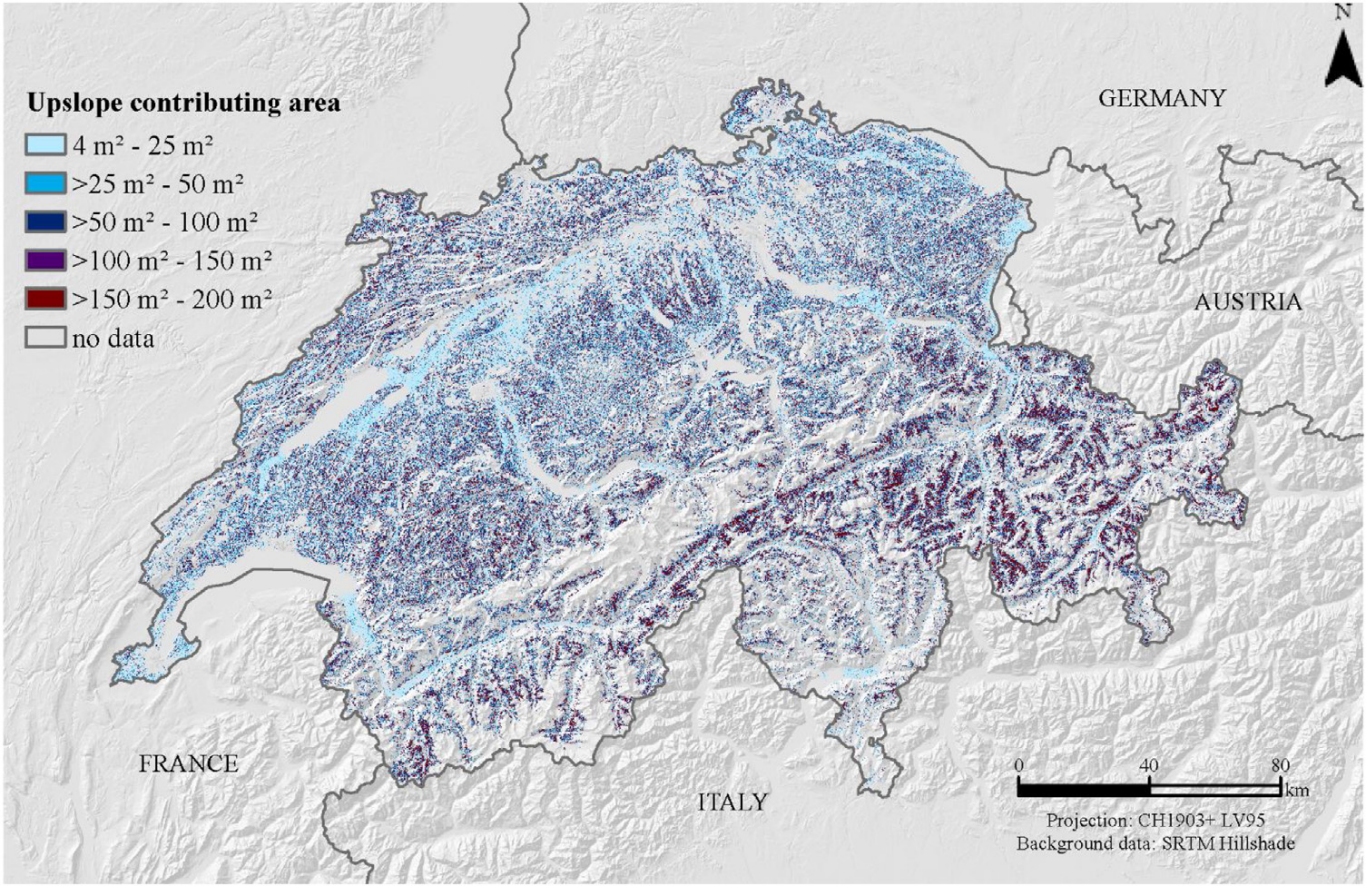
Constraint flow accumulation grid with a maximal flow path length of 100 m.

For our calculation of L-factor using a 2 m resolution Digital Elevation Model, the maximal flow length of 100 m, corresponds to a threshold of 50 cells multiplied by the cell size of 2 m (Fig. 2).

Additionally, maximal flow path length was constrained by a field block cadaster. The cadaster defines hydrological units of continuous agricultural land, that are separated by landscape elements acting as flow boundaries (e.g., forests, streets, urban areas, water bodies, or ditches) following the approach of Winchell et al. [14].

### Proposed adaption of the S-factor

In 2014, we conducted a total of 16 rainfall simulations on alpine slopes to assess the soil loss rates related to different slope inclinations (Table 2; [17]). The experiments were conducted at a north and south facing slope both with grassland cover in the mountains of the Urseren Valley, Switzerland. At each slope two transects were selected with slope gradient ranging from 20 to 90%. We used a field hybrid rainfall simulator modified after Schindler Wildhaber et al. [18] with an intensity of 60 mm h^−^1, which is comparable to a high rainfall event in this area.

**Table 2:** Rainfall simulation measurements at the two study sites on steep alpine slopes in Switzerland under consideration of different inclinations and vegetation cover.

N^o^	inclination (°)	vegetation cover (%)	measured sediment rate (t ha^−1^ yr^−1^)	normalized^a^ sediment rate (t ha^−1^ yr^−1^)	normalized^a^ sediment rate without outliers (t ha^−1^ yr^−1^)
1	17	23	13.8	8.5	8.5
2	22	33	0.6	0.7	0.7
3	11	27	0.0	0.0	0.0
4	27	41	1.2	1.6	1.6
5	31	35	0.2	0.2	0.2
6	35	34	6.8	5.6	5.6
7	42	53	9.4	19.0	19.0
8	39	26	31.0	17.4	17.4
9	11	33	0.6	0.7	0.7
10	17	36	1.4	1.8	1.8
11	22	47	1.3	2.0	2.0
12	27	33	34.3	40.6	
13	31	63	26.1	111.3	
14	35	38	11.1	13.1	13.1
15	39	34	40.2	26.0	26.0
16	42	40	75.4	69.8	

a By C-factor with 35% vegetation cover, L-factor of 1.2, and K-factor of 0.031.

**Fig. 3:**
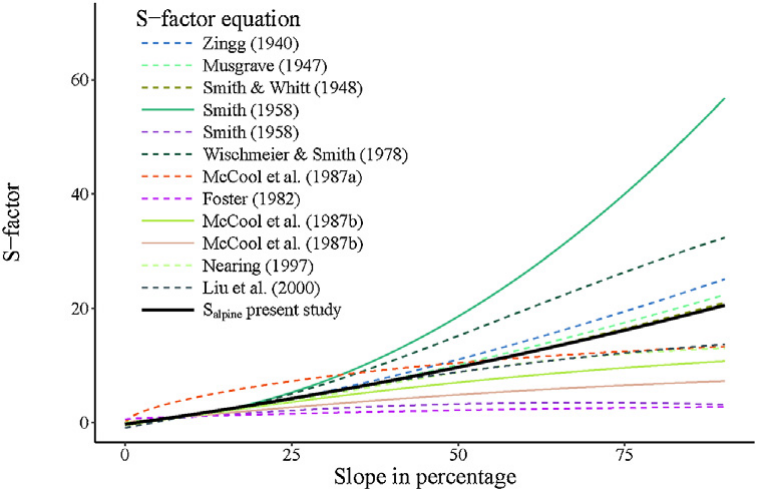
Review and behavior of different empirical S-factor functions and the fitted function for steep alpine environments (S_alpine_).

The experimental sites showed small variation in vegetation cover, soil erodibility, and slope length (due to the effect of slope angle), therefore all experimental plots were normalized to average values of the respective factors. S-factors were fitted to observed soil loss versus sine of the slope angle using an exponential, power, and polynomial equation to the original dataset with all observation and a dataset excluding one outlier (N° 13), and three outliers (N° 12, 13, 16). The nine regression lines yield R² estimates between 0.18 and 0.70, but differ largely with increasing slope steepness. This range of S-factors with increasing steepness is comparable to previous developed empirical S-factor equations (Table 1, Fig. 1). Therefore, we decided that a fitted function (S_alpine_ in Table 1, Fig. 3) complying the most important S-factors from the literature would be most suitable to describe the soil loss behavior at steep slopes. The aggregated S function and is a quadratic polynomic function with progressive growth (Eq. (9)):

(9) $$S_{alpine}=0.0005s^{2}+0.7956s-0.4418$$

where s is the slope steepness in percent.

S_alpine_ is very close to the empirical normalized function proposed by Musgrave [6] for a slope steepness of 9%.

## The Swiss LS-factor map including the Alps

**Fig. 4:**
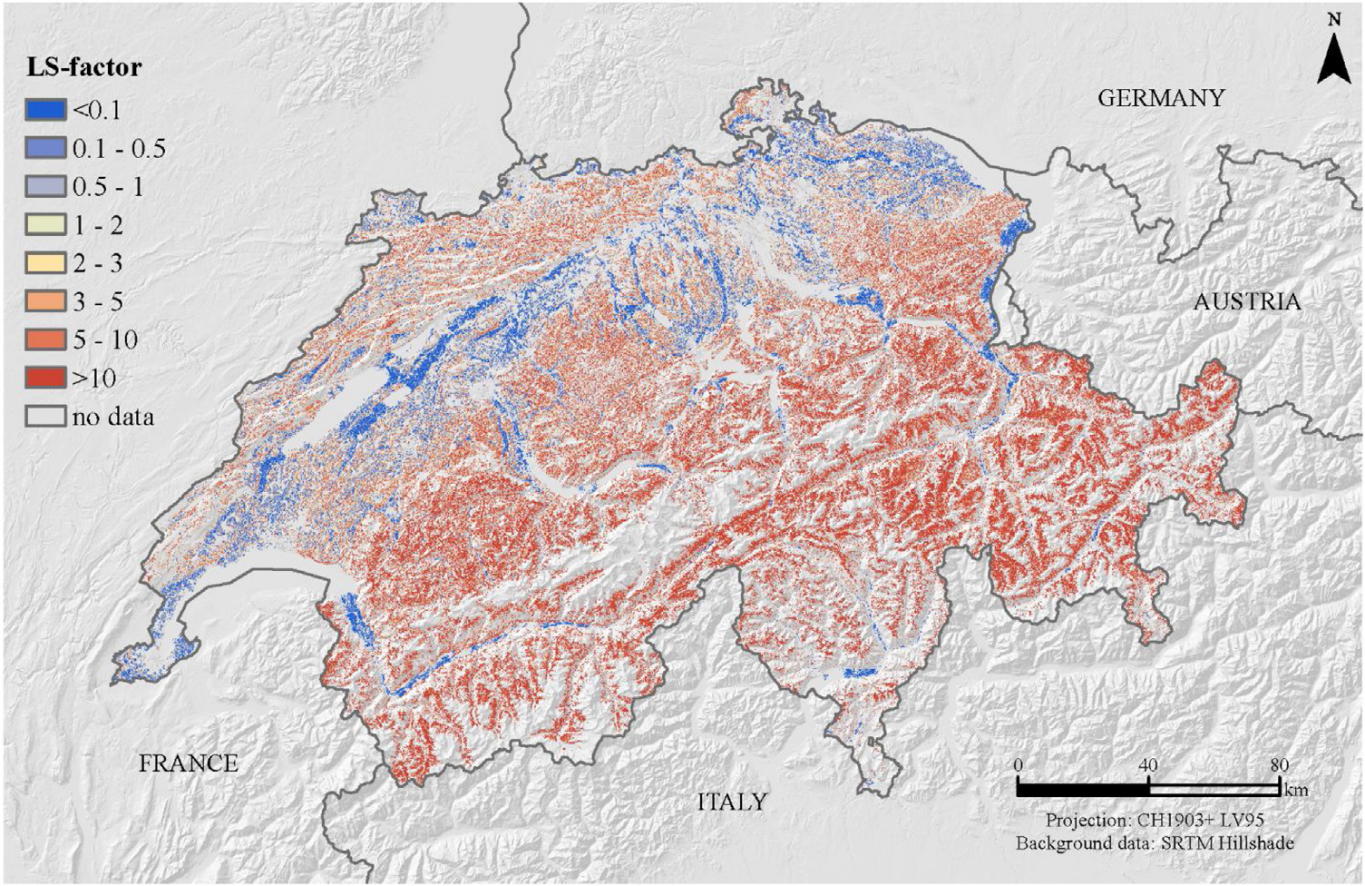
LS_alpine_-factor map (spatial resolution 2 m) for Switzerland derived by the digital elevation model SwissAlti3D.

The resulting modeled mean LS_alpine_-factor of Switzerland is 14.8. The LS-factor increases with elevation gradient from a mean of 7.0 in the zone <1500 m a.s.l. to 30.4 in the zone >1500 m a.s.l. A cluster of highest mean LS-factors can be found across the Alps (Fig. 4). The lowest mean LS-factors are in the Swiss lowlands. South-western facing slopes have higher LS-factors (17.6) compared to plain surfaces (0.04) and north facing slopes (12.5).

### Quality assessment and method uncertainties

The original LS-factor has its origin in empirical field experiments and is developed for a maximum slope steepness of 50%. Validation of existing equations for slopes that are steeper than 50% is a challenge. However, while previous studies at inclinations >25% with approximately 20 plot measurements ([19], 24 plots; [20], 19 plots; [12], 9 plots; [21], 22 plots; [18], 6 plots) were successful in delineating and S-factor equation, in our case the variability of the data impeded a unique solution of the S-factor equation. To account for this high variability and still existing uncertainty, the way forward is to include the variability in the LS-factor calculation.

We investigated the deviation in percentage of our proposed S_alpine_ to a conservative function and a rather progressive function. The conservative function (S_cons_) is based on the translated and scaled sine functions of Eqs. (5) and (6) by McCool et al. [3] with a proportional and slightly digressive growth. The progressive function (S_prog_) is a quadratic polynomic function according to Smith and Whitt [7] with a progressive growth, but a higher coefficient than the here presented fitted function S_alpine_ (Eq. (10)) for S_alpine_.

(10) $$S_{prog}=0.00650s^{2}+0.0453s+0.065$$

where s is the slope steepness in percent.

Low uncertainty has a deviation close to 0%. Higher percentages equals to a higher deviation of S_cons/prog_ to S_alpine_.

**Fig. 5:**
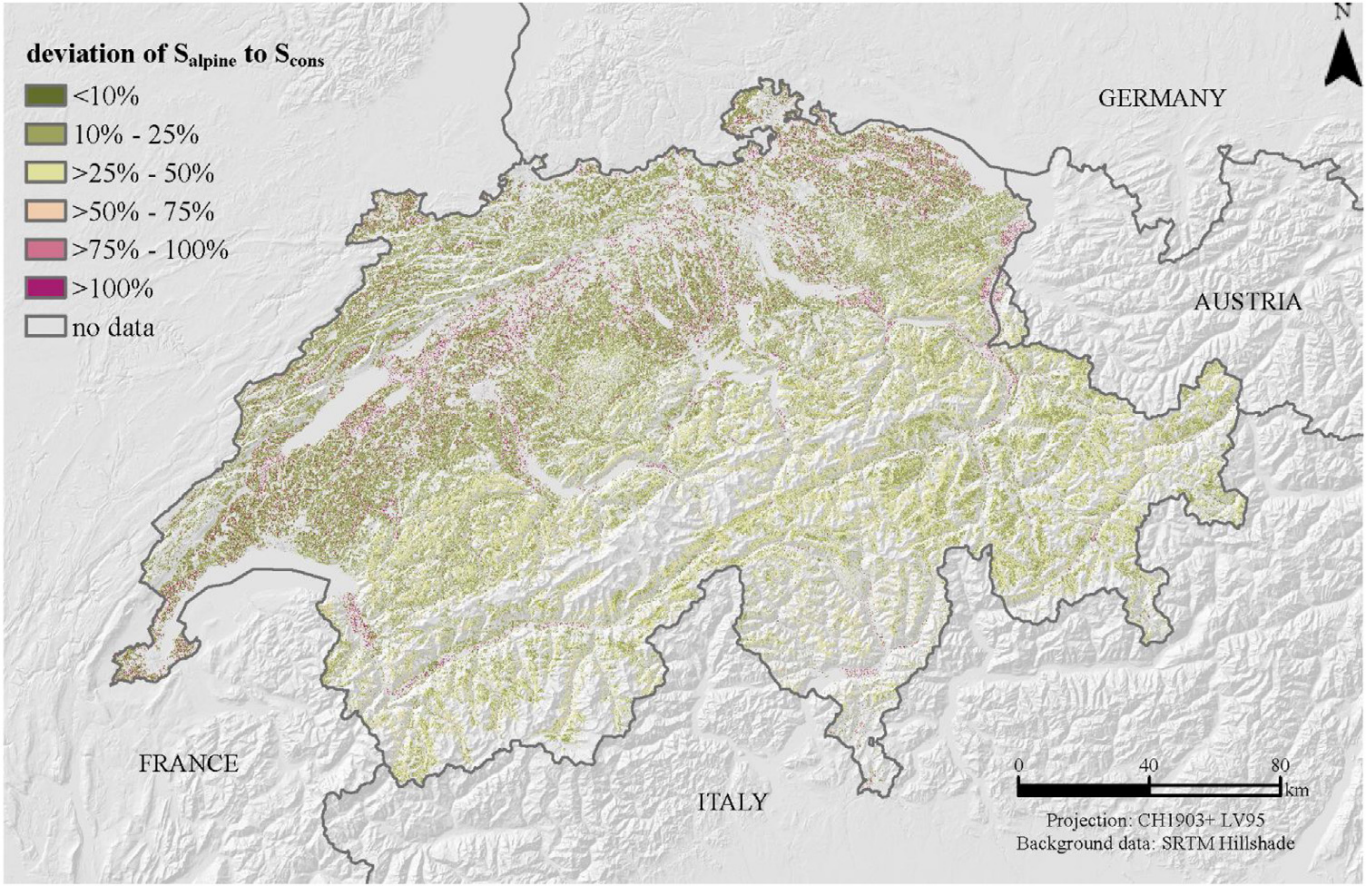
Deviation in percentage of S_alpine_ to S_cons_ as an indicator of quality for the proposed S_alpine_-factor. S_alpine_ is a lumped S-factor of a total of 12 empiric S-factor equations of the literature (Eq. (9)). It can be seen as an approximation to the high slope gradients in alpine environments. S_cons_ complies with the proposed S-factor of McCool et al. [3] (Eqs. (5) and (6)). The deviation is presented in percentage.

**Fig. 6:**
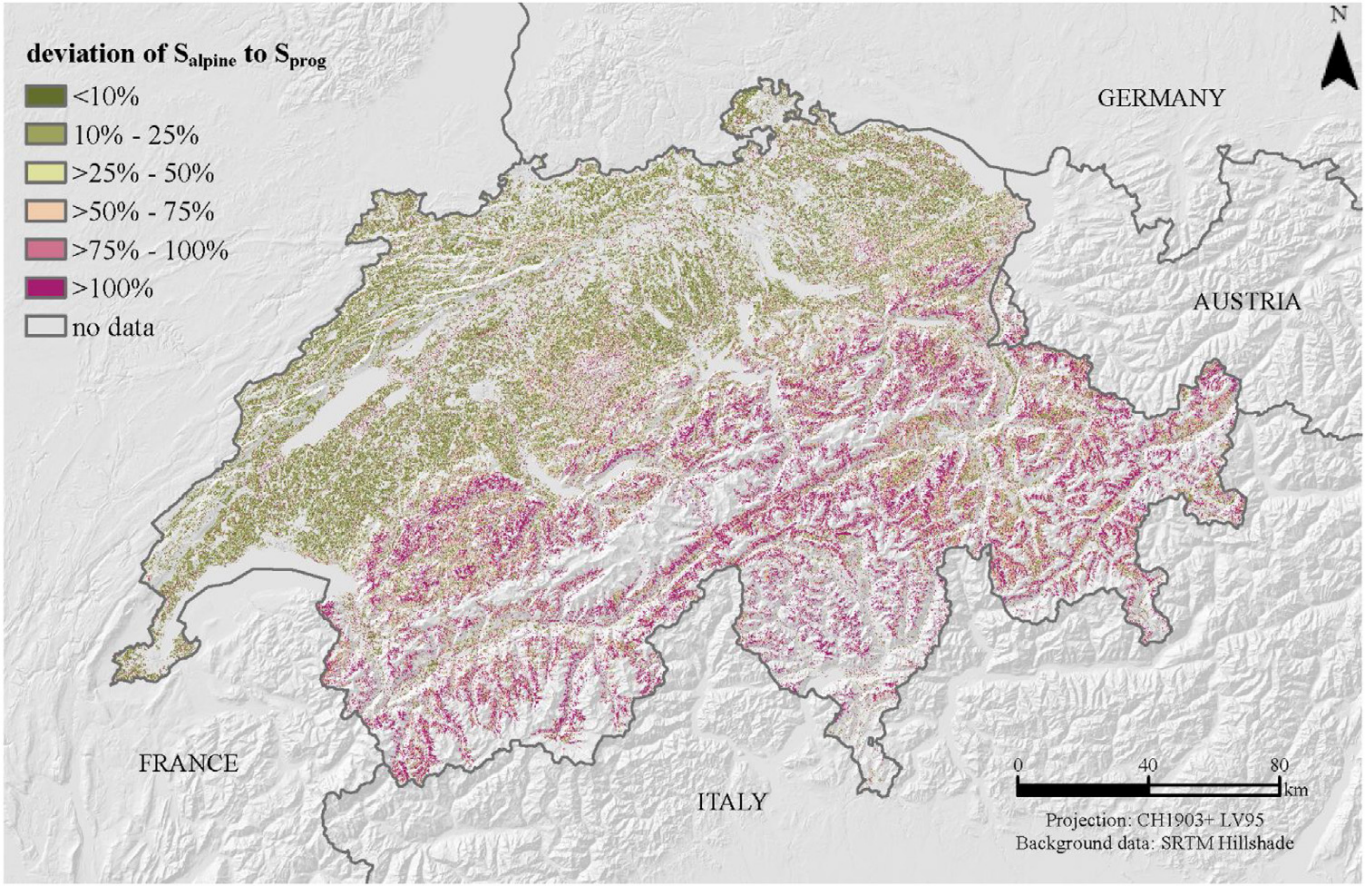
Deviation in percentage of S_alpine_ to S_prog_ as an indicator of quality for the proposed S_alpine_-factor. S_alpine_ is a lumped S-factor of a total of 12 empiric S-factor equations of the literature (Eq. (9)). It can be seen as an approximation to the high slope gradients in alpine environments. S_prog_ complies with the proposed S-factor of Smith and Whitt [7] (Eq. (10)). The deviation is presented in percentage.

The deviation of S_alpine_ to S_cons_ shows higher deviations in areas with less slope gradiants (parts of Swiss midland) (Fig. 5). The steep slope areas in the Alps have deviations of 25%–50%. Both functions, S_alpine_ and S_cons_ predict the steep alpine environment in a comparable way. The deviation of the progressive S-factor (Sprog) and S_alpine_ diverge much more in the Alps whereas the equations are rather fitting in flatter regions (Fig. 6). A sharp edge of low divergence to high divergence is marked by the northern Alpine foothill with increasing slope gradients.

This relationship of deviation and slope gradient is not surprising as the uncertainty of many equations rises with slope steepness (cf. Fig. 3). García-Ruiz et al. [22] identified an increasing trend of uncertainty for 624 measured erosion rates and slope gradients across the world for slope steepness >11°.

**Fig. 7:**
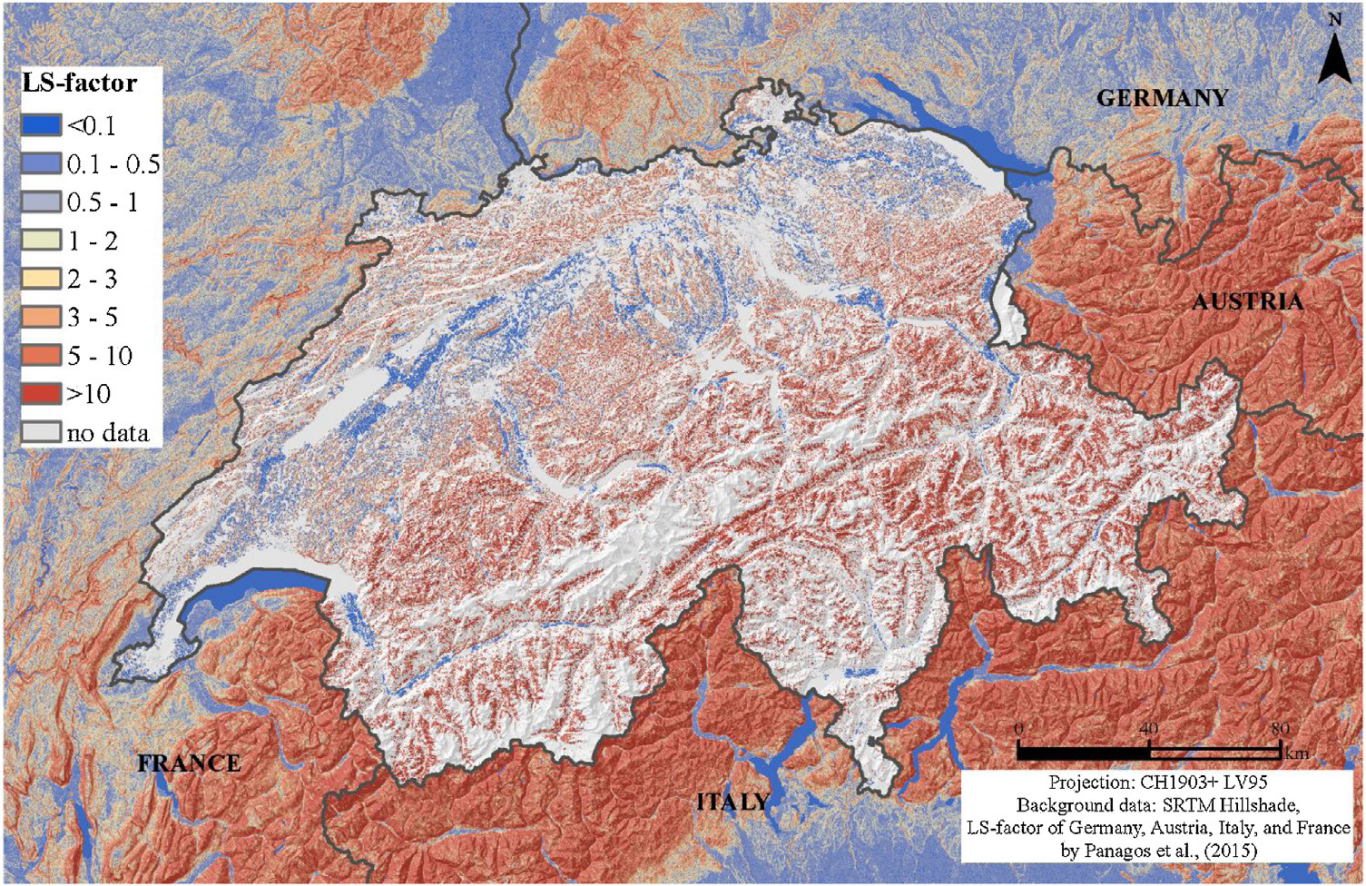
LS-factor for the Swiss agricultural area (incl. Liechtenstein) embedded in the European Union’s LS-factor map (for total country area) by Panagos et al. [23].

The LS-Factor map of the Swiss agricultural land use unit is visually compatible with the LS-factor maps of the European Union provided by Panagos et al. [23] (Fig. 7). In contrast to the modeling of the total country area by Panagos et al. [23] we constrained the LS-factor to agricultural soils incl. grasslands using a field cadaster. The main differences are found on steeper slopes >50%, which have been excluded in the European approach. Furthermore, the European map relies on the conservative Eqs. (5) and (6) by McCool et al. [3]. Additionally, different spatial resolutions of Digital Elevation Models (2 m versus 25 m) are influencing the slope and aspect mapping and thus the LS-factor [[24], [25], [26]].

It should be considered that the number of rainfall experiments for the L-factor (n = 19) and the S-factor (S = 16) is short and limited only to grasslands which are the predominant land use at Swiss alpine slopes [27]. Rainfall simulations in alpine environments are difficult to conduct due to the harsh terrain and climate conditions. Often, the temporal period for measurements is limited by the late melt out of snow cover and the short vegetation period [28]. To better model the S-factor for steep alpine slopes further measurements (e.g., rainfall simulation experiments) are needed to constrain S-factor assessment for steep slopes.

## Additional information

### Introduction

The slope length factor L and slope steepness factor S, often lumped together as the topographic factor LS. The LS-factor is one of the factors (R rainfall erosivity, C cover and management factor, K soil erodibility, P support practices) of the Universal Soil Loss Equation (USLE) and its revised version (RUSLE) [1,29]. LS is a factor that describes the influence of the topography to the soil erosion risk by considering the length of a slope and the influence of surface runoff which can be active on eroding soil material before it infiltrates or continuous as interflow. Furthermore, it includes the steepness of a slope as runoff on steeper slopes has a higher gravity and therefore is more relevant for erosion.

With the availability of Digital Elevation Models the calculation of LS-factors in GIS environments was made possible even for large-scale erosion modeling approaches. Winchell et al. [14] revealed a reasonable agreement of GIS-based LS-factor and field measured LS-factors of the US Natural Resource Inventory database for the Mississippi Catchment.

Originally, the LS-factor was assessed on a 9% steep slope with a length of 22.13 m (72.6 feet) [1]. Owing to its empirical character, LS-factors are usually limited to a maximum slope angle of 50% (26.6°) [3,12]. As Switzerland is a country with a high elevation gradient from 192 m a.s.l. to 4633 m a.s.l. (mean elevation 1288 m a.s.l.) and a mean slope gradient of up to 36% (20°), a not negligible fraction of slopes (4.7%) exceeds the limitation of 50%. Yet, no uniform equation to assess the LS-factor for steep slopes like in the alpine environment of Switzerland was presented to the scientific community. Only a few studies are dealing with LS-factors on steep slopes (e.g. [12]). For example, slopes >50% were disregarded in the most recent European Union’s LS-factor map by Panagos et al. [23].

To overcome that limitation in LS-factor modeling on steep slopes, we (i) limited the potential flow path length to a maximal flow and (ii) choose the most representative equation for Swiss steep slopes.

## Data statement

Raw data were generated at Swisstopo and provided only for scientific purposes. Derived data supporting the findings of this study are available from the corresponding author SS on request.
